# Signal dependent ER export of lemur tyrosine kinase 2

**DOI:** 10.1186/s12860-015-0072-6

**Published:** 2015-11-11

**Authors:** E. C. Butler, Neil A. Bradbury

**Affiliations:** Department of Physiology and Biophysics, Chicago Medical School, 3333 Green Bay Rd, North Chicago, IL 60064 USA

**Keywords:** LMTK2, Di-acidic, ER export, Transferrin, Recycling

## Abstract

**Background:**

The membrane anchored kinase, LMTK2, is a serine/threonine kinase predominantly localized to endosomal compartments. LMTK2 has been shown to be involved in the trafficking of the CFTR ion channel, the androgen receptor, as well as modulating neurodegeneration. As a membrane anchored protein, LMTK2 must be exported from the ER, yet the mechanisms whereby LMTK2 is sequestered within the ER for efficient export are unknown.

**Methods:**

Sequence analysis of the carboxyl tail of LMTK2 revealed a putative di-acidic ER export motif. Site-directed mutagenesis was utilized to ablate this potential motif. Subcellular fractionation, immunofluorescence microscopy, and transferrin recycling assays were used to determine the consequence of mutating LMTK2’s export motif.

**Results:**

Mutation of the di-acidic export motif led to ER retention of LMTK2, and an increase in protein half-life and a concomitant loss of LMTK2 from its appropriate terminal destination. Loss of LMTK2 from endosomal compartments by preventing its release from the ER is linked to a reduction in transferrin recycling.

**Conclusions:**

We have identified a di-acidic ER export motif within the carboxyl tail of the membrane anchored kinase LMTK2. This sequence is used by LMTK2 for its efficient export from the ER.

## Background

Irrespective of the final destination, all integral membrane proteins are initially synthesized on ribosomes associated with the rough endoplasmic reticulum (ER). The first step in the exit of newly synthesized proteins from the ER, for subsequent trafficking to intacellular organelles or the cell surface, is the incorporation of nascent proteins is their incorporation into COP II transport vesicles. Initiated by the binding of GTP to the small GTPase Sar1, the association of other coat proteins (COPs) Sec23 and Sec24, forms a pre-budding complex that yields a concave surface enriched in basic residues [1–3]. Initially thought to be a bulk flow process [4], the efficient export of proteins from the endoplasmic reticulum (ER) is now thought to be a selective process [5], whereby the Sar1-Sec23/24 complex not only elicits membrane curvature, but also selectively enriches cargo proteins into the newly forming COP vesicle. Several groups have identified specific amino acid sequences within the cytoplasmic domains of newly synthesized (and properly folded) proteins that act as called ER export motifs [6–8]. One identified ER export motif consists of two adjacent bulky hydrophobic or aromatic residues, as seen in the FF motif of ERGIC53 [9] or the VV motif in the NMDA receptor [2]. The second class of ER export motifs is characterized by two acidic residues separated by a variable amino acid ([D/E]x[D/E]). Initially identified in the vesicular stomatitis virus glycoprotein (VSVG) (^502^TDIE) [10]. this motif is now found in other mammalian proteins, including potassium channels (KAT1, ^495^DTE; TASK-3, ^252^EDE ^257^DAE) [11–13] the cystic fibrosis transmembrane conductance regulator (CFTR; ^565^DAD) [14, 15] and the angiotensin II receptor (^357^EME) [16]. Of note for VSV-G, an upstream tyrosine residue appears to enhance the activity of the di-acidic motif (^501^YTDIE) [10].

**Fig. 1:**
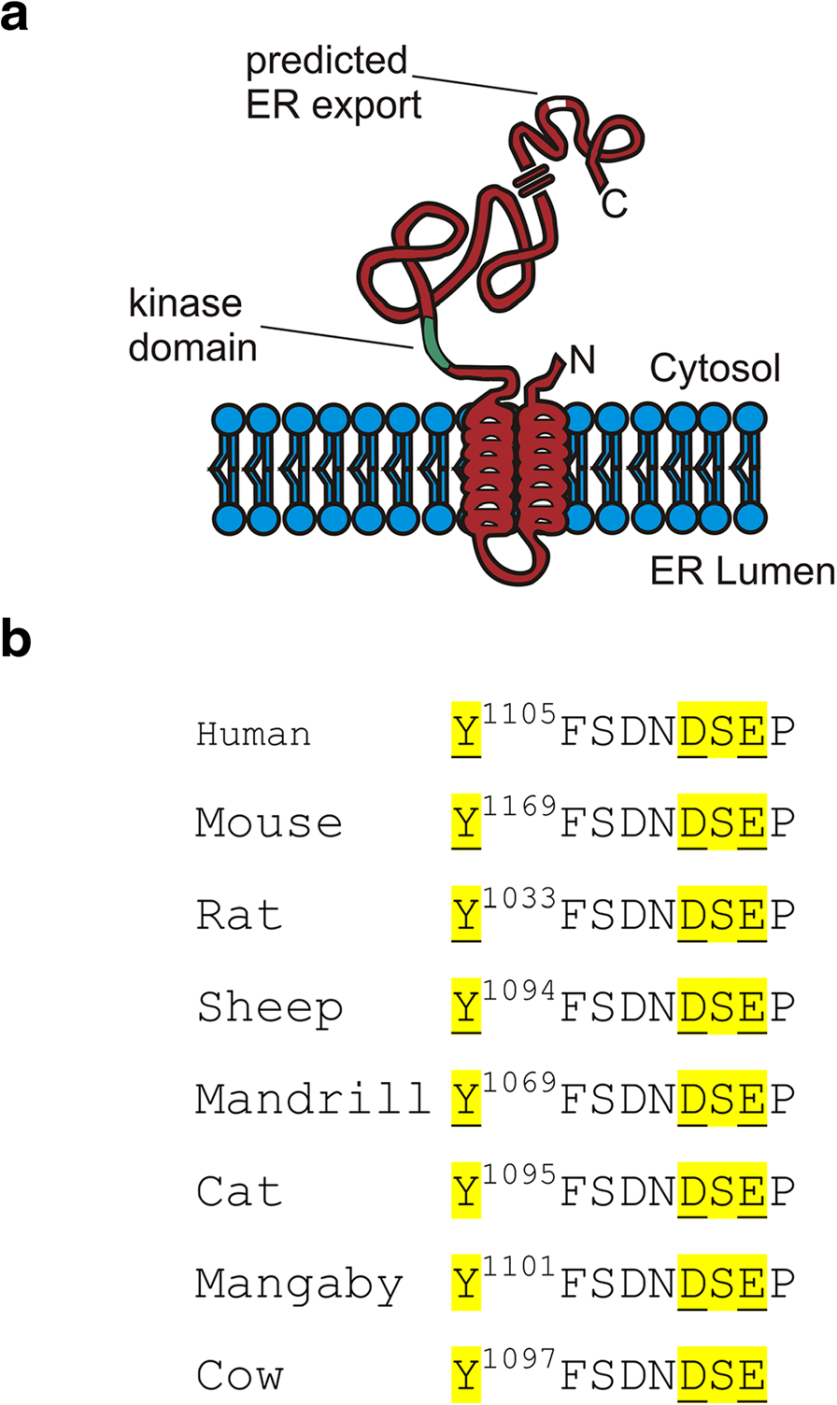
Topology and location of the predicted ER export motif in LMTK2. **a** The kinase domain (green) and predicted ER export motif (*white*) of LMTK2 are located on the cytosolic side of the ER membrane. The location of the ER export motif in the cytosol places it to interact with COPII vesicle components (adapted from [29]. **b** Alignment of the C-termini of LMTK2 orthologs from different species. The Di-acidic motif is shown in yellow. All sequences were obtained from NCBI website and aligned using VectorNTi software

Lemur tyrosine kinase 2 (LMTK2), also known as BREK, AATYK-2, cdk5, KPI-2 and Cprk [17, 18] is a membrane anchored 1503 amino acid serine/threonine kinase. LMTK2 is not highly expressed in the plasma membrane, but rather is present on intracellular vesicles, where it has multiple functions depending upon the cell type in which it is expressed. Interestingly, although the molecular pathways between tissues differ, LMTK2 appears to play a role in regulating intracellular trafficking events. For example LMTK2 modulates the trafficking and surface expression of the cystic fibrosis ion channel, CFTR, in airway cells [19, 20]. Similarly LMTK2 modulates the intracellular trafficking of the androgen receptor in prostate epithelia, where LMTK2 negatively regulates the expression of androgen dependent genes [21, 22]. LMTK2 has also been shown to be involved in differentiation of tissues, where LMTK2 plays a role as a negative regulator of NGF-induced neuronal differentiation [17, 23]. Male LMTK2 knockout mice are infertile due to lack of development and differentiation of gonadal tissue [17, 24]. Several binding partners have been identified for LMTK2, including myosin VI [25, 26], including PP1C [18] and cdk5/p35 [27]. It is likely that LMTK2 works in concert with myosin VI to modulate the trafficking events observed in various tissues. Moreover, the fact that LMTK2 is a kinase, and can also modulate the actions of phosphatases [18, 20, 28], argues that LMTK2 may also be an important mediator, integrating several intracellular signaling and trafficking pathways. LMTK2 contains two hydrophobic domains, which anchor the kinase in lipid bilayers, with a short amino and long carboxyl tail that reside in the cytosol [29, 30]; it is within the long carboxyl tail that the catalytic activity of the kinase resides (Fig. 1a). Although there is a clear role for LMTK2 in regulating endocytosis and recycling through its presence in endosomal membranes [17, 25, 26], the pathways and mechanisms by which nascent LMTK2 exits the ER and enters endosomes is not known. The initial step in the trafficking of any membrane protein to its final destination is its efficient export from the ER. To this end, our results suggest that the di-acidic motif is a key determinant in the export of LMTK2 from the ER. Subsequently, it is likely that other, as yet undetermined, signals will be required for the final targeting of LMTK2 to its final endosomal destination.

## Methods

### Materials

CHO and HeLa cells were purchased from ATCC (Manassas, VA). DMEM, Advanced DMEM, DMEM/F12, Penicillin, Streptomycin, Geneticin, Lipofectamine 200 and Alexa546-conjugated transferrin were all obtained from Life Technologies (Gaithersburg, MD). pCI vectors were from Promega (Madison, WI). Fluorescent constructs were obtained from OriGene (Rockville, MD) and AddGene (Cambridge, MA). Antibodies used were LMTK2 (Rabbit monoclonal, Sigma Aldrich, St Louis MO; note the antibody recognizes a region distal to the putative di-acidic motif [22, 29]), TGN46 (Rabbit Polyclonal, Abcam, Cambridge MA), EEA1 (Rabbit monoclonal, Cell Signaling, CA), PDI (Rabbit polyclonal, Santa Cruz, CA), GAPDH (rabbit polyclonal; Santa Cruz). Secondary antibodies were goat anti-rabbit IRDye 680RD conjugates (Licor, Lincoln, NE), and all images were collected on a Licor Odyssey SA imager. For immunofluorescence microscopy, labeled secondary antibodies (cy3 and alexa 488 conjugates) were from Jackson Laboratories (West Grove, PA). All other reagents were obtained from Sigma and were of reagent grade.

### Cell culture

CHO Cells [31] were maintained in DMEM/F12 (Life Technologies), and HeLa cells in Advanced DMEM (Life Technologies). All media was supplemented with 10 % FBS and 1 % penicillin/streptomycin, and cells grown in 5 % CO_2_ at 37 °C. Cell transfections were performed using Lipofectamine 2000™ according to the manufacturer’s protocol. Selection of stable cells was achieved by growing cells in media supplemented with 500 μg/ml geneticin for LMTK2 constructs, 100 μg/ml neomycin for mEmerald-TfR, and 3 mg/ml puromycin for LMTK2 siRNA.

### Constructs for LMTK2

shRNA against human LMTK2 was from Origene (Rockvile, MD) and contained a puromycin mammalian selection marker for stable selection. Human LMTK2 was a generous gift from Dr Tadashi Yamamoto (Tokyo University) and was expressed in the pCI vector. The LMTK2 A^1110^SA mutant (D^1110^ASE^1112^A) was generated using site-directed mutagenesis using the QuikChange II XL Site-Directed Muagenesis Kit (Agilent, Santa Clara, CA). shRNA resistant constructs were generated by introducing 4 silent mutations into the coding regions of wt and A^1110^SA LMTK2, that overlapped with the recognition sequence of the shRNA construct. LMTK2 constructs were transiently expressed.

### Immunofluorescence micoscopy

Cells growing on coverslips to ~60 % confluence were fixed with 4 % paraformaldehyde, permeabilized with 0.2 % Triton X-100, blocked with 1 % BSA in PBS and processed for indirect immunofluorescence using primary antibodies as described in Figure Legends. Secondary antibodies coupled to Alexa-Fluor-488 were used to visualize binding of the primary antibodies. Cells were visualized with an Olympus FluoView F10i confocal microscope (Olympus, Tokyo, Japan). For transferrin uptake experiments, cells were starved in RPMI media containing 0.2 % BSA for 2 h, loaded with 5 μg/ml Alex-Tf (Sigma) in RPMI-0.2 % BSA for 20 min at 37 °C, washed in PBS and fixed. To calculate a Pearson’s coefficient, multiple randomly selected fields from several experiments were collected, and ImageJ software (NIH, Bethesda MD) used to evaluate signal overlap.

### Immunoblot analysis

Cells were lysed in TGH buffer (1 % Triton X-100, 10 % glycerol, 25 mM Hepes-Na, pH 7.4) and resolved by SDS-PAGE as previously described [29]. GAPDH was used as a loading control. Detection of primary antibody binding was performed using IRDye-conjugated secondary antibodies (LiCor Biosciences) followed by visualization on an Odyssey SA infrared imaging system (LiCor Biosciences).

### FACS analysis of transferring recycling

Transferrin-uptake and recycling was performed as previously described [32]. CHO cells stably expressing the human transferrin receptor (TfR; to bind and internalize human transferrin) were subject to transient transfection with either wt or A^1110^SA LMTK2 . Briefly, cells were incubated with transferrin coupled to Alexa-Fluor 555 (Tf-Alexa-Fluor-555; Sigma) for 30 min at 4 °C followed by internalization at 37 °C in the continuous presence of Tf-Alexa-Fluor 555). Cells were then washed and incubated at 37 °C in media supplemented with 100 μg/ml unlabeled transferrin for 30 min before fixation with 3.7 % PFA. Cell associated Tf-Alexa-Fluor 555 was determined by FACS analysis using a BD LSRII flow cytometer (BD Biosciences).

### Subcellular fractination and organelle enrichment

Enrichment of major intracellular organelles was performed using a self-generated iodixanol (OptiPrep™) density gradient. Briefly, cells were homogenized in a sucrose-containing detergent-free buffer at 4 °C using and Dounce-type homogenizer to maintain organelle integrity. The homogenate was subject to a series of low speed centrifugations to remove unbroken cells, nuclei, mitochondria and lipids to generate a post mitochondrial fraction. This fraction was adjusted to 20 % (w/v) OptiPrep and layered between 30 and 15 % (w/v) OptiPrep cushions. Samples were subject to centrifugation (3 h at 150,000 x *g*) at 4 °C. Fractions were collected from the top using a syringe and 25G needle. Collected fractions were solubilized in Laemmli sample buffer and resolved by SDS-PAGE on a 4-12 % Bis-Tris gradient gel (Life Technologies). Proteins were transferred to PVDF membranes and probed with appropriate antibodies.

## Results

### LMTK2 contains potential acidic ER export motifs

Alignment of the sequences of various mammalian orthologs of LMTK2 revealed the presence of a canonical di-acidic motif in the distal portion of the C-terminal tail (Fig. 1a, b), irrespective of the overall length of each species LMTK2. Interestingly, as with the di-acidic export motif in VSV-G, a proximal upstream tyrosine residue is also seen. To explore the possible role acidic amino acids in the efficient export of LMTK2 from the ER, we studied the trafficking of wild-type (wt) and mutant LMTK2 (^A1110^SA), where we mutated the D^1110^SE of sequence of wild-type LMTK2 to A^1110^SA.

### Co-localization of LMTK2 mutants with ER Markers

**Fig. 2:**
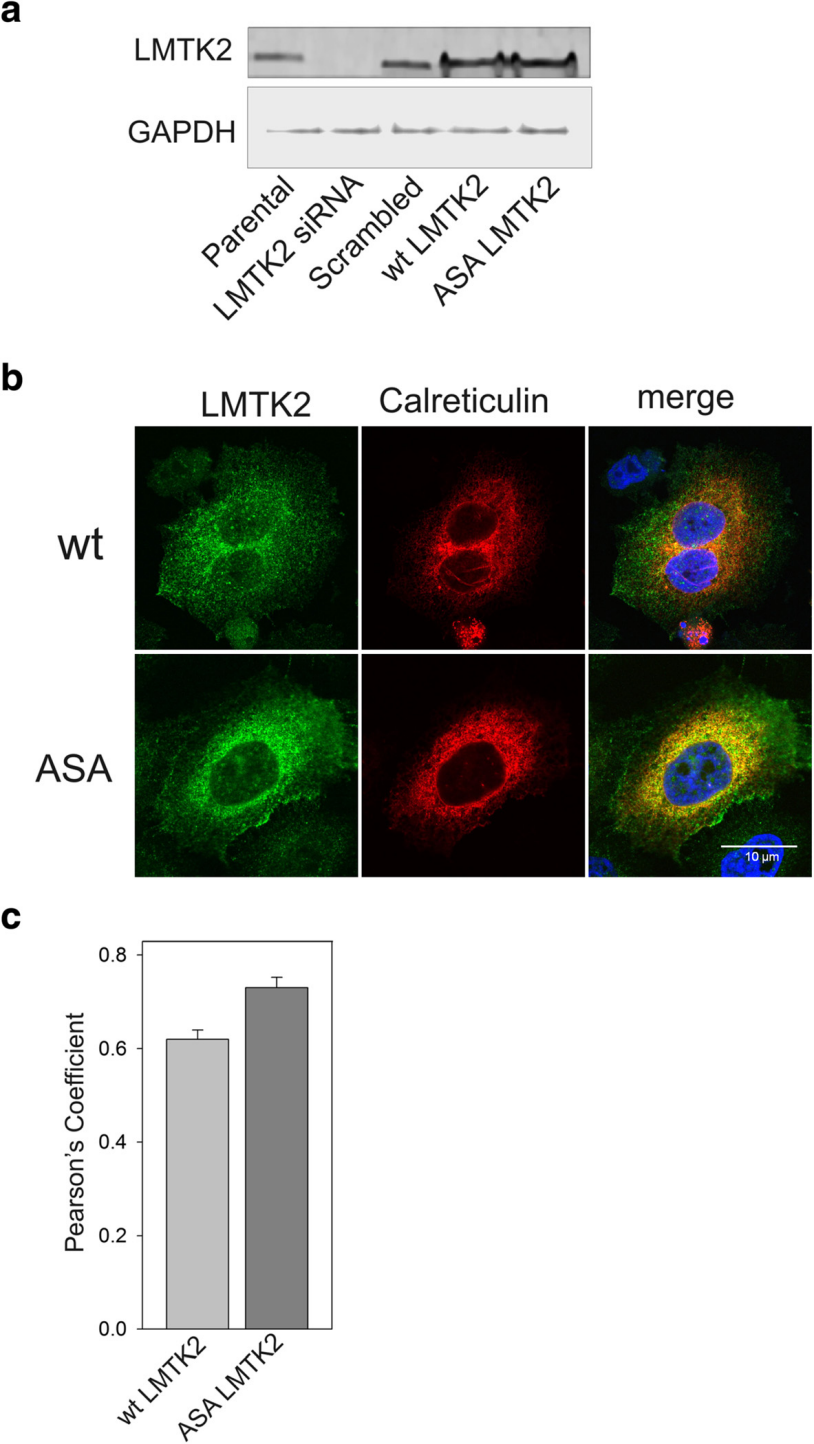
Mutation of the di-acidic export motif causes retention of LMTK2 within the ER. **a** HeLa cells stably expressing shRNA against LMTK2 were transiently transfected with siRNA resistant constructs for either WT LMTK2 or A1110SA LMTK2. **b** Following immunofluorescence labeling, LMTK2 was visualized with secondary antibodies conjugated to Alexa 488 dyes (*green*) and calreticulin observed as the transiently expressed mRFP fusion protein (*red*). Nuclei are stained blue using DAPI. Bar = 10 μm. **c** Pearson’s coefficients were determined for HeLa cells by imaging at least 30 cells from three independent experiments, encompassing randomly selected fields of view. *p < 0.01 for difference by Student’s *t-test*

To investigate whether mutation of putative acidic ER export signals affects the subcellular distribution of LMTK2, Hela cell lines expressing either wt or A^1110^SA LMTK2 were evaluated. Endogenous LMTK2 was knocked down using a stable shRNA against LMTK2 (Fig. 2a), scrambled shRNA had no effect. shRNA revealed a knockdown of ~80 % of endogenous LMTK2. Transient expression of shRNA resistant wt and A^1110^SA LMTK2 revealed expression of both constructs. Overexpression of shRNA resistant wt LMTK2 was performed to rule out any expression artefacts, and to be able to more accurately compare wt and A^111^0SA LMTK2 localization. Determination of the location of the endoplasmic reticulum was performed by transient co-expression of mFRP calreticulin (Fig. 2b). Wt LMTK2 signal was observed in discrete puctate structures towards the periphery of the cells, with some signal also seen in the perinuclear region. In contrast, A^1110^SA-LMTK2 displayed a more prominent perinuclear staining pattern, with reduced peripheral vesicular staining. Confirmation that the perinuclear staining overlapped with the endoplasmic reticulum, was determined by observing co-localization of A^1110^SA LMTK2 with the ER marker calreticulin. A Pearson co-efficient analysis revealed a significantly higher co-localization (*p < 0.01*) between A^1110^SA LMTK2 and calreticulin, compared to wt LMTK2 and calreticulin, arguing for reduced ER export of the A^1110^SA mutant compared to wt LMTK2.

**Fig. 3:**
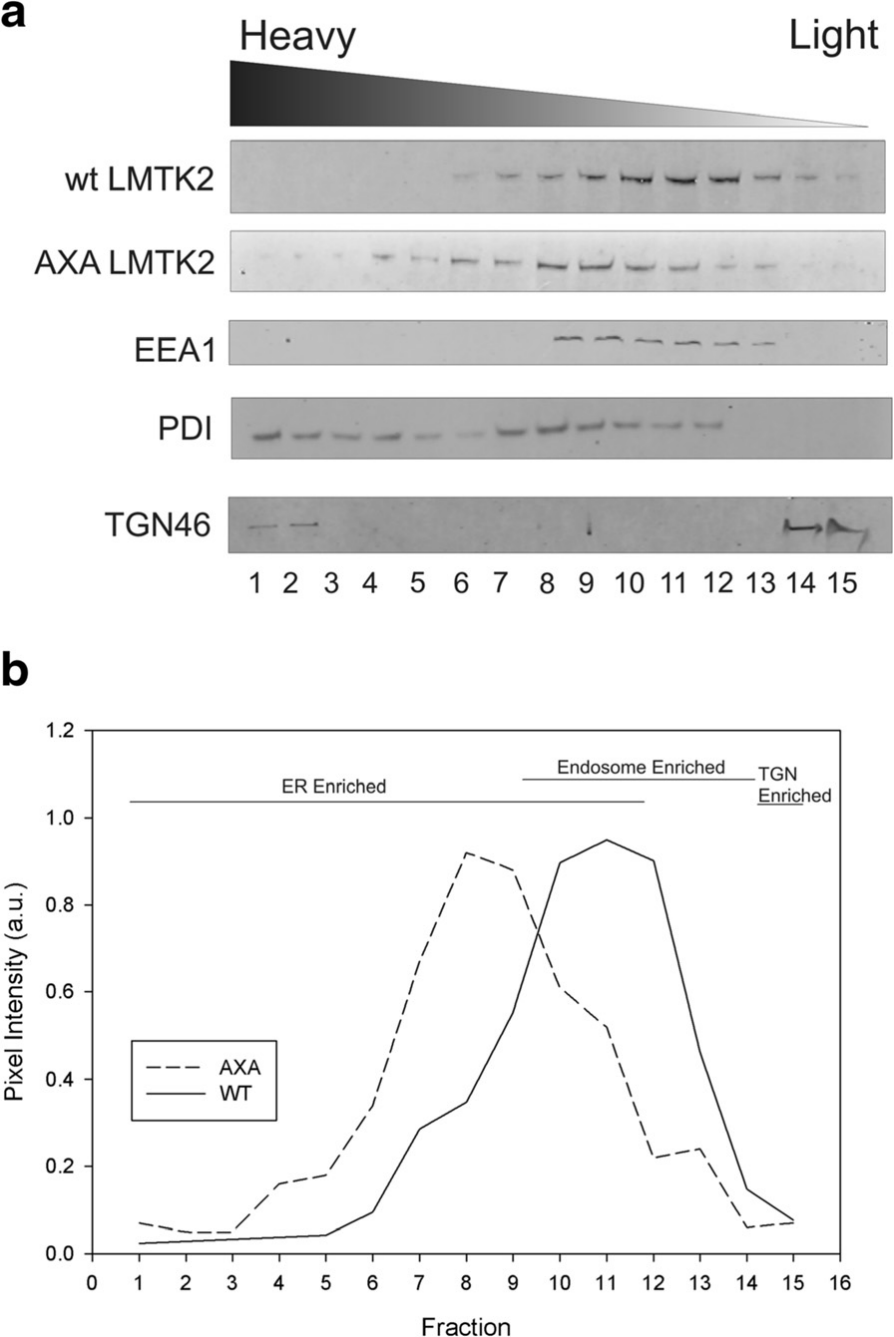
Gradient fractionation of cellular organelles shows a retention of A1110SA LMTK2 within the ER. HeLa cells expressing either WT LMTK2 of A1110SA LMTK2 were subject to homogenization in a detergent free buffer, using a Dounce-type homogenizer. Post-nuclear supernatants were applied to an Optiprep™ gradient and resolved by centrifugation. **a** Fractions were collected and proteins resolved by SDS-PAGE on a 4–12 % gradient gel prior to immunoblotting. **b** Comparison of relative band intensities of LMTK2 in each fraction (mean data from 4 separate experiments)

To further elucidate the relative subcellular distributions of wt and A^1110^SA LMTK2, and to evaluate ER retention of the A^1110^SA mutant, cells expressing wt or A^1110^SA LMTK2 were subject to homogenization in a Dounce-type homogenizer in detergent-free buffer, to retain organellar integrity. Post nuclear crude microsomes were subject to separation on a self-generating iodixanol (Optiprep™) gradient medium. Fractions containing enriched subcellular organelles were subject to immunoblot analysis. Several antibodies for ER markers (PDI), Golgi (TGN46), and endosomes (EEA1) were used to probe the various fractions, as well as antibodies against LMTK2. Immunoblot analysis of fractions from the Optiprep™ gradients revealed wt LMTK2 to be found predominantly towards the lighter fractions, with signal also co-fractionating with endosomal markers (Fig. 3). A small signal was also identified in denser ER fractions, consistent with the synthesis of LMTK2 as a membrane protein synthesized within the ER. In contrast to WT LMTK2, A^1110^SA LMTK2 displayed a shift in buoyant density away from lighter regions towards heavier and denser fractions. This was seen as a reduction in LMTK2 content from endosomal fractions and a greater preponderance of A^1110^SA LMTK2 in ER enriched fractions compared to wt LMTK2. No large steady-state levels of LMTK2 in TGN enriched fractions were observed.

**Fig. 4:**
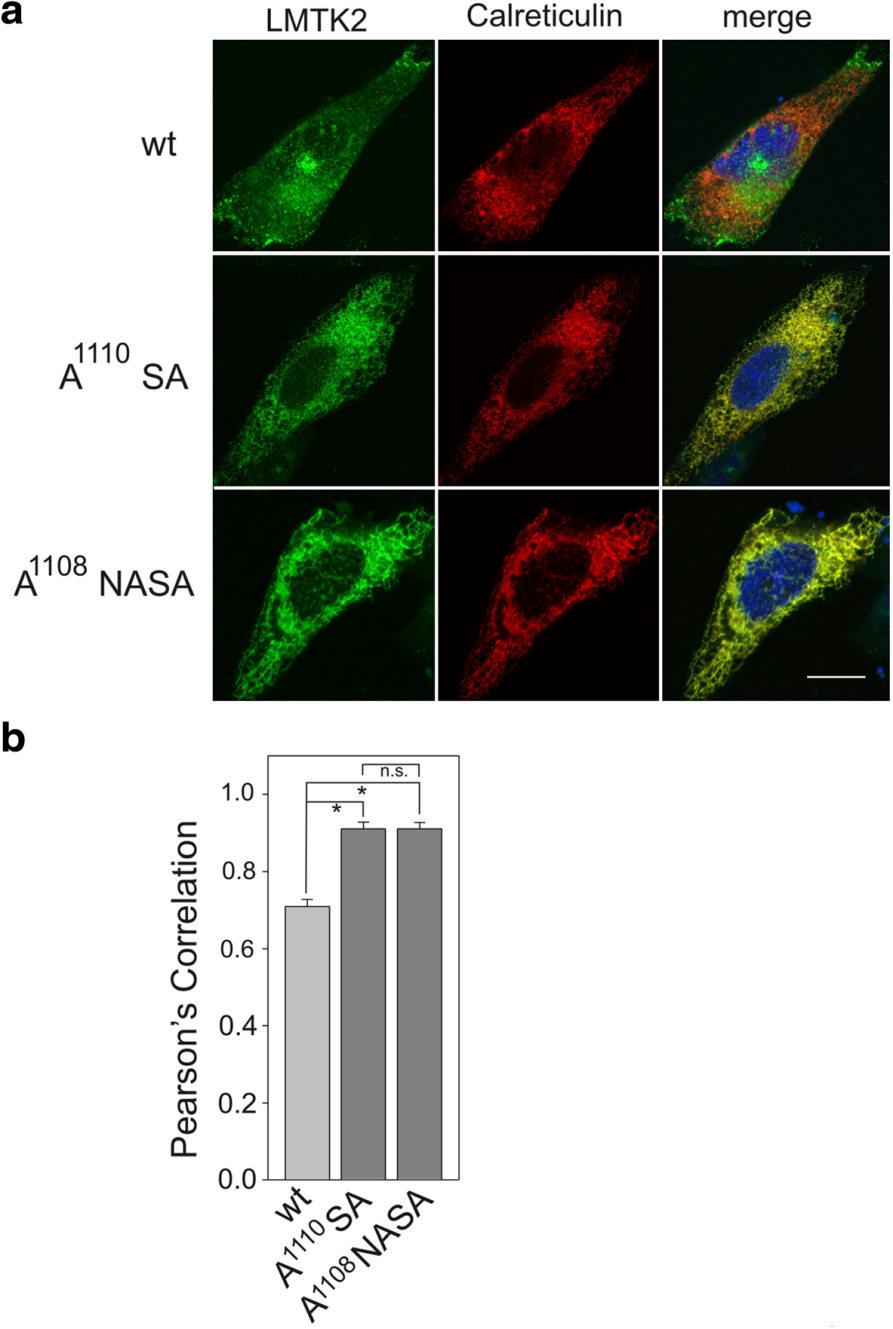
Lack of evidence for a tri-acidic export signal for LMTK2. **a** Cells stably expressing either WT, di-acidic mutants (A1110SA) or tri-acidic mutants (ANA1110SA) of LMTK2 (*green*), along with mRFP calreticulin (*red*), were fixed and imaged using immunofluorescence miscroscopy. Nuclei are stained blue using DAPI. Bar = 10 μm. **b** Pearson’s coefficients were determined for cells by imaging at least 30 cells from various randomly selected fields of view. *p < 0.01 for difference by Student’s *t-test.* n.s. = not significantly different

One study evaluating the role of acidic export motifs has suggested a tri-acidic sequence to be present in the inwardly rectifying potassium channel, KAT1 [11]. Since there is an acidic amino acid upstream of D^1110^ at position D^1108^, we evaluated the possible role of this amino acid by constructing a triple Asp → Ala construct (D^1108^NDSE → A^1108^NA1110SA). Transient expression of WT, A1110SA and ANA1110SA LMTK2 was performed and co-expressed with mRFP-calreticulin (Fig. 4a). As before, WT LMTK2 was found in discrete punctate structures extending past the ER into the cytoplasm. In contrast, both A1110SA and ANA1110SA LMTK2 were observed predominantly in perinuclear regions, which overlapped with calreticulin staining. Peason’s coefficient analysis (Fig. 4b) showed a significantly greater overlap between A1110SA and ANA1110SA LMTK2 compared to WT LMTK2 (*p < .001 n* = 30 cells for each condition). When the overlap of calreticulin with A1110SA and ANA1110SA LMTK2 was compared however, no significant difference in localization between the two mutants was observed (*p >0.8*; *n* = 30 cells for each condition).

### Mutation of acidic export motifs increases the half-life of LMTK2

**Fig. 5:**
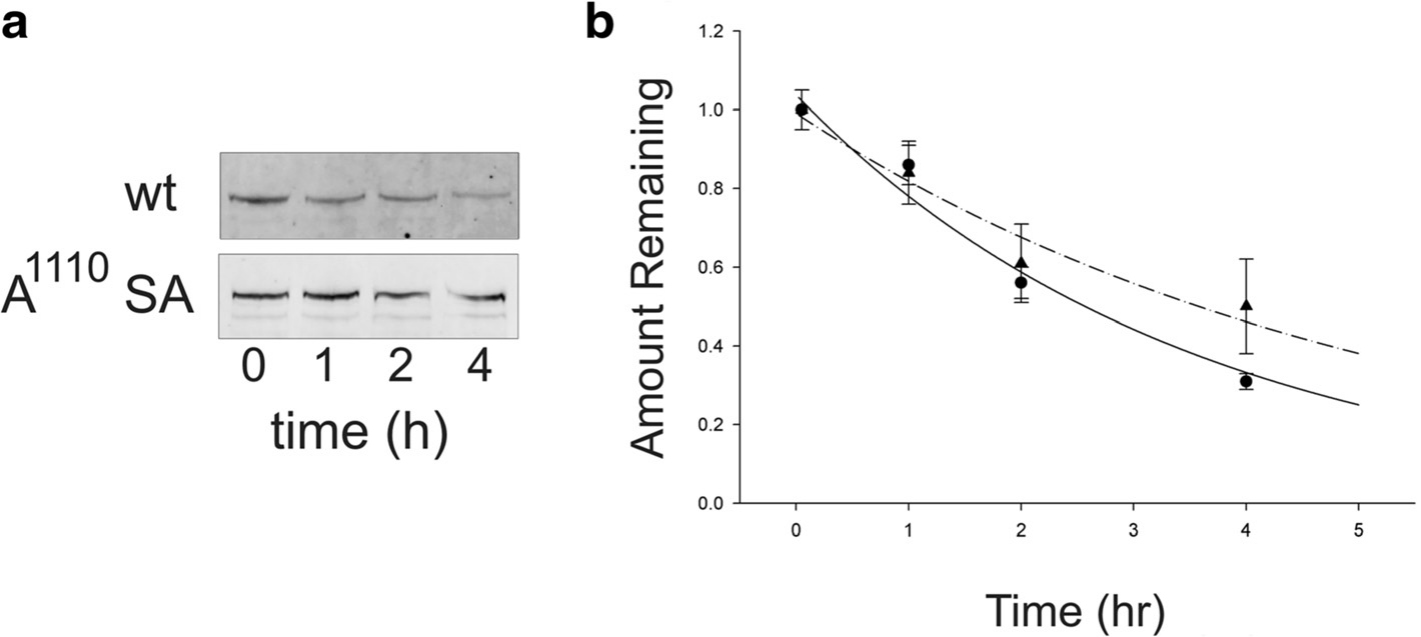
Effect of ER export motif mutations on protein half-life. **a** Cells expressing either WT or A1110SA LMTK2 were subject to labeling with ^35^S Met/Cys to steady-state. Cells were switched to ^35^S-free media and sampled at various time-points. LMTK2 was subject to immunoprecipitation and resolved by SDS-PAGE. A typical gel is shown. **b** Analysis of three separate experiments yields half-lives of 2.5 h for WT LMTK2 (●) and 3.5 h for A1110SA LMTK2 (▲) (*p < 0.05* for difference between constructs)

Pulse-chase experiments were performed to determine the kinetic effects of the A1110SA mutation on LMTK2 degradation, and the life-time of the protein once it is formed (Fig. 5). Both proteins exhibited fairly short half-lives with t_1/2_ for both constructs being less than 5 h. Interestingly, the A^1110^SA mutation yielded a protein that exhibited a small but significant (*p <0.05*) increase in the stability of the protein, with wt LMTK2 having a half-life of ~2.5 h and A^1110^SA LMTK2 having a half-life of ~3.5 h.

### Mutation of acidic export motifs alters transferrin recycling

**Fig. 6:**
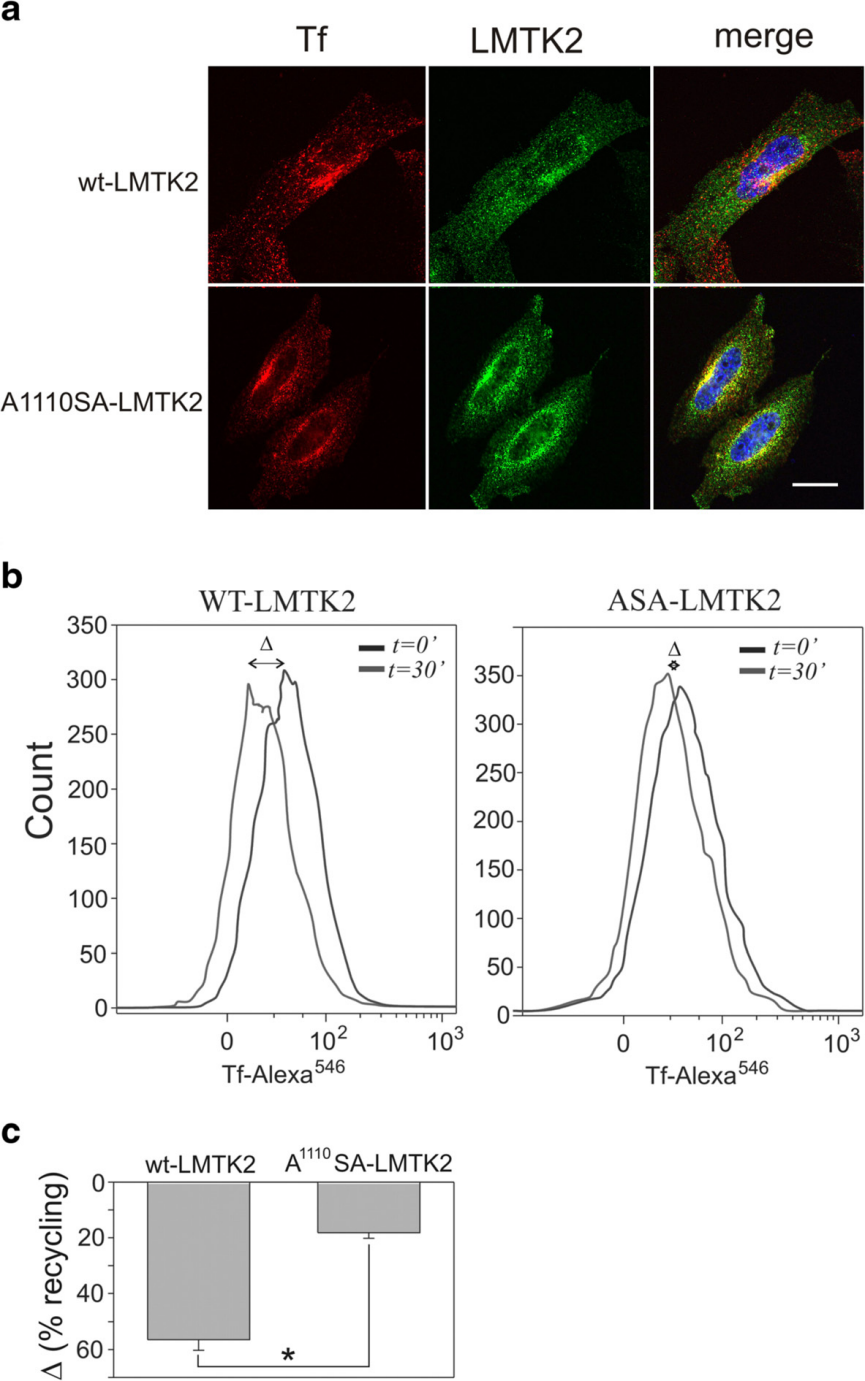
Loss of LMTK2 ER export impairs transferrin recycling. **a** Cells were loaded with Tf-Alexa-Fluor 555 before fixation. Images are confocal z-projections. Bar 10 μm. **b** Cells were starved of transferrin in serum free media for 30 mins, followed by exposure to fluorescently labeled transferrin (Tf-Alexa 546) for 30 mins at 4 °C, followed by an additional 30 mins at 37 °C. Cells were rinsed in Tf-Alexa 546 free media supplemented with unlabeled transferrin, and stained using antibodies against LMTK2 (*green*). Red (Tf Alexa 546), blue (nuclei stained with DAPI). **c** Flow cytometry shows the total amount of transferrin taken up by cells at time *t = 0* min (grey rectangle) and time *t = 30* min chase (white rectangle). Data are mean ± SD from three independent experiments. *p <0.05 for difference between wt and A^1110^SA LMTK2

It has been previously shown that LMTK2 is involved in endosomal recycling [25]. We hypothesized that ablation of the ER export signal for LMTK2 would reduce LMTK2 levels within endosomal membranes and yield a similar phenotype to LMTK2 knockdown. To test this hypothesis, cells were pulsed with fluorescently labeled transferrin to facilitate uptake of transferrin into endosomal components of the endosomal recycling complex (ERC). The ERC is a perinuclear collection of tubule-vesicular membranes containing Rab11 [33, 34]. CHO cells were utilized due to the absence of detectable LMTK2 [26], which despite knock down in HeLa cells was still present. To evaluate the functional effects of LMTK2 ER exit on transferring recycling, in the absence of a potentially confounding LMTK2 background, we elected to express wt and A^1110^SA in CHO cells, along with the human transferrin receptor. To follow the uptake of transferrin in wt and A^1110^SA LMTK2 expressing cells, cells were pulsed for 20 min with fluorescently labeled transferrin (Alexa-546 transferrin, Molecular Probes). As shown in Fig. 6a, in WT-LMTK2 expressing cells, internalized transferrin is present in the cell periphery, but also smaller amounts within a perinuclear region. In contrast, in A^1110^SA-LMTK2 expressing cells, transferrin was trapped within a swollen perinuclear recycling compartment. We used FACS analysis to quantify the intracellular trafficking of transferrin in wt-LMTK2 and A^1110^SA-LMTK2 expressing cells (see details in Materials and Methods). To determine if loss of LMTK2 from its appropriate cellular location inhibited recycling of transferrin we pulsed cells with fluorescently labeled transferrin, followed by a chase of 30 min in the presence of excess unlabeled transferrin to facilitate recycling. Both wt- and A^1110^SA-LMTK2 expressing cells took up transferrin to similar levels (Fig. 6b). However, comparing the remaining amount of transferrin in cells, following a 30 min chase, revealed a small but consistent defect in transferrin recycling in A^1110^SA-LMTK2 expressing cells (Fig. 6b, c).

## Discussion

The role of LMTK2 in cells is just beginning to be unraveled, but appears to play important roles in ion channel trafficking [19], androgen signaling in prostate cancer [22] and neuro-degeneration [23]. We have previously shown that the long carboxyl tail, as well as the short amino terminus, of LMTK2 are located within the cytosol [29, 30]. Moreover, LMTK2 appears to be an important binding partner of other trafficking and signaling molecules, including myosin VI [25], protein phosphatase 1/inhibitor 2 [18], CFTR [19], and the androgen receptor [21, 22]. Given these manifold functions and accessory proteins, it is clear that the appropriate subcellular localization of LMTK2 is critical to its cellular physiology. As with all membrane proteins, LMTK2 is initially synthesized within the endoplasmic reticulum, prior to its export within COPII coated vesicles. Rather than bulk flow exit, many proteins contain key regions within their amino acid sequence that facilitate their efficient export from the ER. Di-acidic sequences have been shown to function as ER export motifs in several membrane proteins [10, 12, 15, 35–37]. Among such di-acidic sequences, the DXE sequence is the best characterized, in which both acidic acid residues are required. Comparison of amino acid sequences from LMTK2 orthologs, we identified a stretch of amino acids containing a potential di-acidic export motif that is present in all species of LMTK2 identified. Such amino acid identity conserved across various species is usually indicative of that site playing a key role in the function of the protein. Moreover, the potential di-acidic motif is localized within the cytosolic domain of the protein, where it has the potential to interact with COPII components.

Normally LMTK2 is present within the endosomal compartment of cells, where it regulates trafficking events. Mutation of the di-acidic code at asparate 1110 and glutamate 1112 to alanines caused a marked retention of LMTK2 within the ER, as determined by colocalization with the ER marker calreticulin. We documented such ER retention by both immunofluorescence microscopy and by iodixanol enrichment of subcellular membrane compartments. The finding that ablation of a di-acidic code leads to ER retention of LMTK2 is finding not confined to LMTK2, but also observed in many other proteins bearing a di-acidic ER export motif, including vesicular stomatitis virus protein G [10], G protein coupled receptors [16], potassium channels [38, 39], and anion channels [15]. A small amount of mutant LMTK2 is likely to undergo export from the ER, as the di-acidic signal facilitates efficient ER export, rather than an all or none signal; thus bulk flow export continues unimpeded. We were unable to completely ablate ER export of LMTK2 through mutation of the acidic code, however this is also true for mutation of the di-acidic code in many other proteins, including CFTR [40], the TASK-3 potassium channel [12] and ERGIC-53 [41]. The inability to completely knock out ER export, may be due to several reasons. For example, in addition to the di-acidic code, other signals may also be present in proteins. In several cases, this is due to a tyrosine residue. For example, a conserved tyrosine residue in the transmembrane domain of CASP is needed for its export from the ER to the Golgi [42]. In plants, mutation of the tyrosine residue alone in CASP does not impair ER export, however it can support a degree of ER export in the absence of the di-acidic code [43]. In the present manuscript we did not focus on the potential role of tyrosine residues. In addition, it is unlikely that we were able to completely knock down LMTK2 using shRNA. Thus there is likely some residual endogenous LMTK2 that is still present and confounding our data. Nonetheless, despite the observation that we were not able to have complete colocalization of A^1110^SA LMTK2, our results were nonetheless consistent in showing a significant difference between wt and mutant LMTK2. Moreover, the consistency in our findings using two independent methologies (i.e., immunofluorescence microscopy and subcellular fractionation) supports the notion that our results are valid. Although we have not formally ruled out any major structural changes associated with our mutagenesis, the fact that the EX export motif is considerably downstream to the catalytic domain and membrane spanning regions suggests that the protein may fold appropriately. If this is the case, the protein should not be major substrate for ERAD, and could accumulate rather than being degraded. This being the case, we might anticipate that the overall half-life of the protein would be increased. Indeed, this appears to be the case for the anion channel CFTR, where blockade of its ER export (in this case an entirely wild-type CFTR protein) leads to a small but clear increase in CFTR stability [15].

The functional consequences of our preventing the efficient ER export of LMTK2 are reminiscent of studies reducing endosomal LMTK2 through siRNA mediated knock down of LMTK2 [25]. Thus although A^1110^SA LMTK2 is functionally intact (we have no formal evidence in support of this, but our mutations are in regions distinct from the catalytic domain and protein binding regions) the lack of protein in appropriate endosomal regions, due to ER retention, affects membrane trafficking. Loss of endosomal LMTK2 has no marked impact upon transferrin endocytosis, a finding also seen for LMTK2 knock down [25]. However the efficient recycling and loss of transferrin from the cell is impeded in A^1110^SA LMTK2 expressing cells. Although not a complete loss of transferrin recycling, inhibition of LMTK2 export resulted in a similar degree of transferrin retention as that observed for RNAi knockdown of LMTK2 [25]. Although some studies have suggested that blocking TfR delivery from early endosomes to the ERC might increase a direct fast recycling pathway [44], knock down of total LMTK2 does not induce such a pathway [25]; thus it is unlikely that such a pathway would be induced when endosomal LMTK2 levels are depleted through inhibition of efficient ER export. Whilst the first step in targeting LMTK2 to endosomal compartments of necessity is the export of membrane anchored LMTK2 from the ER, it is likely that other signals (i.e., not a di-acidic motif) are responsible for post ER trafficking of LMTK2 to endosomes; such signals remain to be identified. Thus, the di-acidic motif is likely only required to allow LMTK2 to exit the ER efficiently.

Here, we have established that efficient anterograde tranposer of LMTK2, from the site of its synthesis in the ER to its destination in endosomal compartments, requires an acidic ER export motif for its initial exit from the ER. LMTK2 plays an important role in modulating the physiological function of a number of organs, including the airways [19], prostate gland [22] and brain [17, 27], as well as in the development of various human cancers [22, 45, 46]. As we begin to elucidate the cellular and organismal physiology of LMTK2, LMTK2 may provide a novel target for the treatment of human diseases in which LMTK2 is involved.

## Conclusion

In conclusion, our results provide evidence for the presence of a di-acidic ER export motif in LMTK2, and provide evidence that the export of LMTK2 from the ER occurs through a selective mechanism. Since LMTK2 plays a pivotal role in a number of human diseases, targeting LMTK2 and its trafficking may provide a novel foundation for designing drugs to treat diseases impacted by LMTK2.

## Abbreviations

LMTK2: Lemur tyrosine kinase 2
TfR: Transferrin receptor
